# Enhanced expression of miR-20a driven by nanog exacerbated the degradation of extracellular matrix in thoracic aortic dissection

**DOI:** 10.1016/j.ncrna.2024.05.006

**Published:** 2024-05-20

**Authors:** Zhao An, Yangyong Sun, Xiaodong Yang, Jingwen Zhou, Yongchao Yu, Boyao Zhang, Zhiyun Xu, Yuming Zhu, Guokun Wang

**Affiliations:** a Department of Thoracic Surgery, Shanghai Pulmonary Hospital, Tongji University School of Medicine, Shanghai, China; b Department of Cardiovascular Surgery, Changhai Hospital, Naval Medical University, Shanghai, 200433, China; c Department of Cardiothoracic Surgery, Affiliated People's Hospital of Jiangsu University, Zhenjiang, China

**Keywords:** Thoracic aortic dissection, Extracellular matrix degradation, miR-20a, Tissue inhibitors of metalloproteinase 2, Nanog

## Abstract

Thoracic aortic dissection (TAD) is a life-threatening vascular disease manifested as intramural bleeding in the medial layers of the thoracic aorta. The key histopathologic feature of TAD is medial degeneration, characterized by depletion of vascular smooth muscle cells (VSMCs) and degradation of extracellular matrix (ECM). MicroRNA, as essential epigenetic regulators, can inhibit the protein expression of target genes without modifying the sequences. This study aimed to elucidate the role and underlying mechanism of miR-20a, a member of the miR-17-92 cluster, in regulating ECM degradation during the pathogenesis of TAD. The expression of the miR-17-92 cluster was significantly increased in synthetic VSMCs derived from TAD lesions compared to contractile VSMCs isolated from normal thoracic aortas. Notably, the expression of miR-20a was increased in VSMCs in response to serum exposure and various stimuli. In TAD lesions, the expression of miR-20a was significantly negatively correlated with that of elastin. Elevated expression of miR-20a was also observed in thoracic aortas of TAD mice induced by β-aminopropionitrile fumarate and angiotensin II. Overexpression of miR-20a via mimic transfection enhanced the growth and invasive capabilities of VSMCs, with no significant impact on their migratory activity or the expression of phenotypic markers (α-SMA, SM22, and OPN). Silencing of miR-20a with inhibitor transfection mitigated the hyperactivation of MMP2 in VSMCs stimulated by PDGF-bb, as evidenced by reduced levels of active-MMP2 and increased levels of pro-MMP2. Subsequently, TIMP2 was identified as a novel target gene of miR-20a. The role of miR-20a in promoting the activation of MMP2 was mediated by the suppression of TIMP2 expression in VSMCs. In addition, the elevated expression of miR-20a was found to be directly driven by Nanog in VSMCs. Collectively, these findings indicate that miR-20a plays a crucial role in maintaining the homeostasis of the thoracic aortic wall during TAD pathogenesis and may represent a potential therapeutic target for TAD.

## 1 Introduction

Thoracic aortic dissection (TAD) is a life-threatening disease with high mortality, which is characterized by aortic intimal tearing with an annual incidence of approximately 10/100,000 [1,2]. Risk factors for TAD include biological sex, advanced age, pregnancy, genetic variants, and uncontrolled hypertension, etc. [3]. Currently, there are no effective drugs in clinical use that can prevent the formation, progression, and rupture of TAD. Once TAD occurs, surgical intervention is inevitable, but postoperative mortality remains relatively high, despite continuous improvements in surgical techniques [4]. The pathogenesis underlying TAD development is complex. In addition to genetic mutations, phenotypic transformation of vascular smooth muscle cells (VSMCs) [5], dysfunction of endothelial cells [6], infiltration of immune cells [7], and abnormal interactions between vascular cells [8] have been reported to contribute to the pathological remodeling of the aorta, ultimately leading to TAD. Aortic extracellular matrix (ECM) degradation and medial degeneration are key histopathologic features of TAD, yet the detailed mechanisms remain incompletely understood, which significantly hampers progress in drug development [9]. Therefore, elucidating the pathogenesis of TAD and developing effective conservative treatments are urgent priorities.

ECM is the primary noncellular structure component of the aortic wall, providing structural support for vascular cells and maintaining the aortic mechanical capacity in conjunction with VSMCs [10]. ECM remodeling, including elastin degradation and collagen deposition in the aortic wall, contributes to the development of TAD. It has been reported that the balance between tissue inhibitors of metalloproteinases (TIMPs) and matrix metalloproteinases (MMPs) plays an essential role in the stability of aortic ECM [11,12]. MMP2, a sub-type of MMPs, is crucial in ECM degradation associated with cancer metastasis and aortic dilatation [13]. TIMP2 regulates the activation process of latent form pro-MMP2 on the cell surface, and the imbalance of MMP2-TIMP2 can exacerbate aortic pathological remodeling, ultimately leading to aortic failure [14].

MicroRNAs (miRNAs) are a class of small non-coding RNAs that play essential roles in diverse biological processes. As crucial epigenetic modulators, miRNAs can regulate the expression of target genes by binding to 3′ untranslated region (3’ UTR) without sequence alteration [15,16]. The miR-17-92 cluster is a highly conserved miRNA cluster containing six members (miR-17, miR-18a, miR-19a, miR-19b-1, miR-20a, and miR-92a-1) that collectively participate in a variety of physiological and pathological processes [17]. This study aimed to clarify the role and mechanism of miR-20a, a member of the miR-17-92 cluster, in regulating ECM degradation during the pathogenesis of TAD.

## 2 Materials and methods

### 2.1 Clinical specimens

The aortic specimens were collected during surgical repair of TAD or heart transplantation procedures. Patients with traumatic aortic dissection, Ehlers-Danlos syndrome, Marfan syndrome and other connective tissue disorders, inflammatory aortic diseases, or bicuspid aortic valves were excluded from this study. A total of 26 TAD patients and 10 donor controls were enrolled in this study. Detailed clinical information of the subjects was presented in Table 1. There were no significant differences in the clinical parameters between the two groups.

**Table 1:** Clinical characteristics of the subjects enrolled in this study.

	Control (n = 10)	TAD (n = 26)	*P*
Age [year]	48.9 ± 6.2	48.1 ± 5.0	0.680
Male [n (%)]	8 (80.0)	21 (80.8)	1.000
BMI [kg/m^2^]	22.28 ± 1.35	22.95 ± 1.25	0.163
Smoke [n (%)]	5 (50.0)	9 (34.6)	0.462
Hypertension [n (%)]	3 (30.0)	12 (46.2)	0.468
DM [n (%)]	1 (10.0)	6 (23.1)	0.645
TC [mmol/L]	4.54 ± 1.43	5.09 ± 2.07	0.450
HDL-C [mmol/L]	1.85 ± 0.65	2.08 ± 0.76	0.416
LDL-C [mmol/L]	3.61 ± 0.85	3.99 ± 0.86	0.243
TG [mmol/L]	1.25 ± 0.24	1.16 ± 0.28	0.364
Cr [μmol/L]	85.7 ± 11.7	83.2 ± 16.6	0.665

Data presented as mean ± standard deviation or n (%). TAD, thoracic aortic dissection; BMI, body mass index; DM, diabetes mellitus; TC, total cholesterol; HDL-C, high-density lipoprotein cholesterol; LDL-C, low-density lipoprotein cholesterol; TG, triglyceride; Cr, creatinine.

### 2.2 Animal model

The TAD mouse model was induced by β-aminopropionitrile (BAPN, Sigma-Aldrich, St. Louis, MO, USA) and angiotensin II (Ang II, Sigma-Aldrich) as described previously [18]. Briefly, 3-week-old male C57BL/6J mice (n = 20. SLAC, Shanghai, China) were fed with BAPN dissolved water (1 g/kg per day) for 4 weeks, followed by implantation with osmotic mini pumps (Alzet, Cupertino, CA, USA) filled with 1 mg/kg per minute Ang II for 48 h. Mice in the control group (n = 20) were maintained on a 4-week regular diet and received a 48-h sustained release of normal saline. The thoracic aorta tissues were harvested carefully after humane sacrifice. The animal studies were approved by the institutional review board of Changhai Hospital, and the experiment protocols adhered to the guidelines for the care and use of laboratory animals.

### 2.3 Cell culture

Human aortic VSMCs were isolated from standard aortic specimens as described previously [19]. In brief, aortic specimens were cut into tissue blocks and attached to a culture plate. The plate was then incubated in Smooth Muscle Cell Medium (ScienCell, Carlsbad, CA, USA) at 37 °C with 5.0 % CO_2_. The purity of VSMCs was identified by immunofluorescence of α-smooth muscle actin (α-SMA) and smooth muscle 22α (SM22α). VSMCs between passage 4 and passage 8 were utilized for the experiments.

### 2.4 Quantitative Real-Time polymerase chain reaction (qRT-PCR)

Total RNA was extracted from tissues or cells by TRIzol reagent (Thermo Fisher Scientific, Waltham, MA, USA) according to the manufacturer's instructions. Following RNA quantification using the NanoDrop spectrophotometer (Thermo Fisher Scientific), equal amounts (200 ng) of RNA samples were reverse transcribed to complementary DNA using PrimeScript RT reagent Kit (TaKaRa, Dalian, China) with special stem-loop primer for miRNA and oligo-dT or random primer for mRNA. The expression of miRNA and mRNA was detected by TB Green Premix Ex *Taq*II (Takara Bio) on the LightCycler 480 system (Roche, Basel, Switzerland) following the instructions. RNU6b and GAPDH were amplified as the reference control for miRNA and mRNA detection, respectively. The comparative Ct method was used for result analysis. The primers used in qRT-PCR were detailed in Supplemental Table S1.

### 2.5 Western blot

The expression level of proteins was detected by Western blot assay as described previously [20]. After protein quantification using bicinchoninic acid (BCA) assay (Beyotime, Shanghai, China), equal amounts (200 ng) of protein samples were separated on an SDS‐PAGE gel, followed by transfer to a polyvinylidene difluoride membrane (Pall, Glen Cove, NY, USA). The membrane was blocked with 5 % nonfat milk, then sequentially incubated with the primary and secondary antibodies. Immunoreactive bands were visualized by ECL Plus Western blot analysis Substrate (Thermo Fisher Scientific) on the ChemiDoc MP system (Bio‐Rad, Hercules, CA, USA). GAPDH was used as the loading control. Details of the antibodies was presented in Supplemental Table S2.

### 2.6 Histological and immunohistochemistry

Paraffin-embedded aortic segments were sectioned into 5-μm-thick slides. Following deparaffinization in solvent xylene solution, the slides underwent rehydration in a graded ethanol series. Subsequently, Weigert staining was performed to visualize elastic fibers following the instruction manual (Solarbio, Beijing, China). Immunohistochemistry assays were performed for the detection of target proteins as described previously [21]. Details regarding the primary antibodies were presented in Supplemental Table S2.

### 2.7 Immunofluorescence

The immunofluorescence assay was performed as previously described [22]. Briefly, VSMCs were fixed in 4 % paraformaldehyde and permeabilized with 0.1 % Triton X-100. After blocking by 5 % goat serum, VSMCs were incubated with primary antibodies overnight at 4 °C, followed by incubation with fluorescently labeled secondary antibodies. DAPI was used for the nucleus counterstain. Details of the antibodies were presented in Supplemental Table S2.

### 2.8 Gelatin zymography assay

The activity of MMP2 was assessed by gelatin zymography assay according to the instructions [23]. In brief, protein samples were subjected to electrophoresis on an SDS-PAGE gel co-polymerized with 0.1 % gelatin (Sigma, St Louis, MO, USA) as the substrate. After rinsing in washing buffer containing 2.5 % Triton X-100, the gel was incubated in developing buffer at 37 °C for 36 h, followed by staining with Coomassie Brilliant Blue R250 (Sigma). The activity of MMP2 was presented as unstained bands at about 70 kDa against the opaque blue background.

### 2.9 Gene overexpression and silencing

The mimic and inhibitor of miR-20a, small interfering RNAs (siRNAs) targeting Nanog (si-Nanog) and negative control (si-NC) were designed and synthesized by Ribo Biotechnology Co. Ltd (Guangzhou, China) and transfected into VSMCs using Lipofectamine 3000 reagent (Thermo Fisher Scientific) according to the instruction manual. Detailed information was presented in Supplemental Table S3. Recombinant adenoviruses for Nanog overexpression (Ad-Nanong) and negative control (Ad-GFP) were constructed using the AdMax system (OBiO, Shanghai, China) according to the manufacturer's instructions. The optimal multiplicity of the adenovirus infection in VSMCs was determined to be 25 pfu/cell.

### 2.10 Bioinformatics analysis

Multiple algorithms, including Targetscan, TargetMiner, and miRDB [[24], [25], [26]], were used for target prediction of miRNAs. The potential targets of miR-20a were screened by Venn analysis in FunRich software [27], and was listed in Supplemental Table S4. The complementary matching between miR-20a and 3′-UTR of TIMP2 mRNA was analyzed by RNA-hybrid program [28].

### 2.11 Chromatin immunoprecipitation (ChIP)

The ChIP assay was performed with EpiQuik ChIP Kit (EpiGentek, Farmingdale, NY, USA) following the manufacturer's instructions. At room temperature, VSMCs transfected with Ad-Nanog for 48 h were fixed using 1 % formaldehyde. DNA sonication was completed with a VCX400 ultrasonic processor (Sonics & Materials, Newtown, CT, USA) with the recommended shearing conditions. Immunoprecipitation was performed with an anti-Nanog antibody (Cell Signaling Technology, Boston, Mass, USA), anti-RNA polymerase antibody (positive control), or normal mouse IgG (negative control).

### 2.12 Statistical analysis

Statistical Package for Social Sciences, version 22.0 (Chicago, Ill), was used for data analysis. For categorical variables, statistical differences were evaluated by Fisher's exact test. For continuous variables with equal variances, Students' t-tests and variance analyses following LSD post hoc tests were used for statistical differences analysis between two groups and multiple groups, respectively. For continuous variables with unequal variances, Welch's *t*-test or variance analysis following Tamhane T2 post hoc tests were used for statistical differences analysis between two groups and multiple groups, respectively. Data were presented as mean ± standard deviation, and P < 0.05 was considered statistically significant.

## 3 Results

### 3.1 Expression of the miR-17-92 cluster was upregulated in synthetic VSMCs

**Fig. 1:**
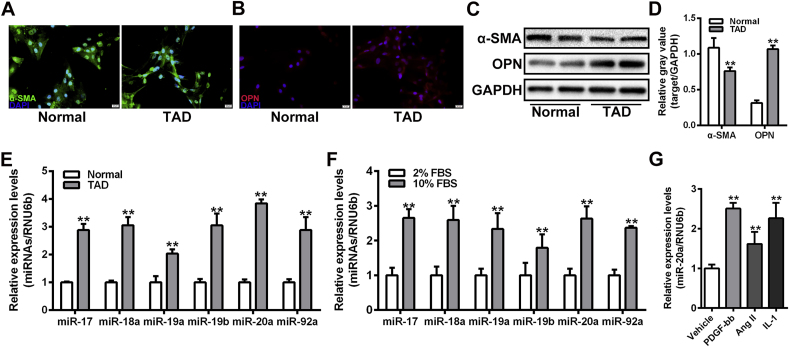
Expression of the miR-17-92 cluster was upregulated in synthetic VSMCs. (A and B) Immunofluorescence assay of α-SMA (green) and OPN (red) in VSMCs isolated from normal and TAD aorta. Nucleus was stained with DAPI (blue). Scale bar = 20 μm. (C and D) Western blot assay of α-SMA and OPN in VSMCs isolated from normal and TAD aorta. The relative gray values of target proteins were evaluated by Image J software. GAPDH was used as a loading control. n = 6 for each group. **P < 0.01. (E) qRT-PCR assay of the expression of miR-17-92 cluster in VSMCs isolated from normal and TAD aorta. RNU6b was used as a loading control. n = 6 for each group. **P < 0.01. (F) qRT-PCR assay of the expression of miR-17-92 cluster in VSMCs cultured in 2 % or 10 % FBS. n = 5 for each group. RNU6b was used as a loading control. **P < 0.01. (G) qRT-PCR assay of the expression of miR-20a in VSMCs stimulated by PDGF-bb, Ang II, or IL-1. RNU6b was used as a loading control. n = 5 for each group.

To investigate the expression profiles of the miR-17-92 cluster in VSMCs with different phenotypes, primary VSMCs were isolated from TAD lesions and normal thoracic aorta, respectively, and identified by immunofluorescence (Supplemental Fig. S1). VSMCs derived from TAD lesions exhibited stronger signals of OPN and weaker signals of α-SMA compared to normal VSMCs (Fig. 1A-B). The decreased expression of α-SMA and increased expression of OPN were further confirmed in VSMCs from TAD lesions by Western blot assay (Fig. 1C-D). Results of the qRT-PCR assay showed that the expression of the miR-17-92 cluster, including miR-17, miR-18a, miR-19a, miR-20a, miR-19b, and miR-92a, was significantly upregulated in VSMCs from TAD lesion (Fig. 1E). Similar expression patterns of the miR-17-92 cluster were observed in VSMCs in response to serum exposure (Fig. 1F). Notably, increased expression of miR-20a was detected in VSMCs upon stimulation with multiple factors, such as PDGF-bb, Ang II, and IL-1 (Fig. 1G).

### 3.2 Elevated expression of miR-20a was associated with ECM degradation in TAD

**Fig. 2:**
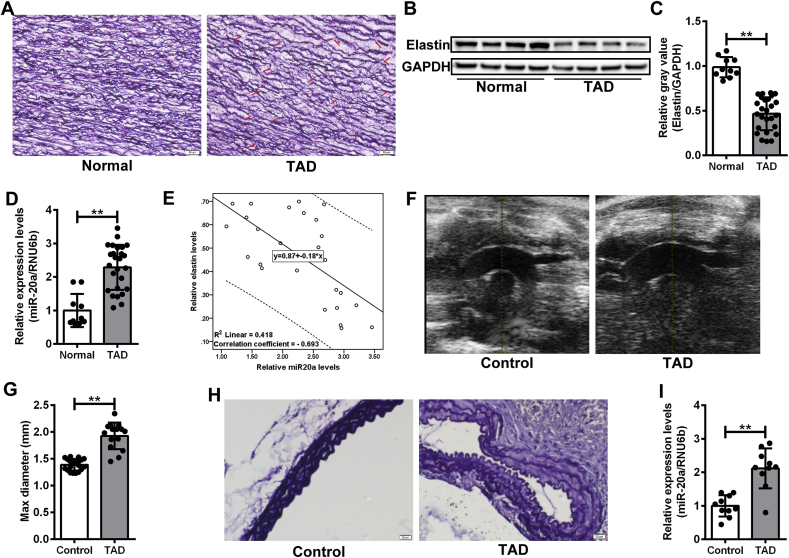
Elevated expression of miR-20a was associated with ECM degradation in TAD. (A) Weigert staining assay of thoracic aorta tissue sections from normal and TAD groups. The breakage of elastic fibers was indicated by a red arrow. Scale bar = 50 μm. (B and C) Western blot assay of elastin expression in thoracic aorta tissues from normal (n = 10) and TAD (n = 26) groups. The relative gray values of target proteins were evaluated by Image J software. GAPDH was used as a loading control. **P < 0.01. (D) qRT-PCR assay of the expression of miR-20a in thoracic aorta tissues from normal (n = 10) and TAD (n = 26) groups. RNU6b was used as a loading control. **P < 0.01. (E) Spearman's rank correlation analysis between miR-20a and elastin expression in the TAD group (n = 26). (F and G) Cardiac echocardiographic imaging of thoracic aorta in mice from Control (n = 20) and TAD (n = 15) groups. The maximum diameter of ascending aorta in the TAD group (1.93 ± 0.25 mm) was significantly larger than that in the control group (1.39 ± 0.11 mm). **P < 0.01. (H) Weigert staining assay of thoracic aorta tissue sections in mice from Control and TAD groups. Scale bar = 20 μm. (I) qRT-PCR assay of the expression of miR-20a in thoracic aorta tissues from normal and TAD groups. RNU6b was used as a loading control. n = 10 for each group. **P < 0.01.

To investigate the potential association between miR-20a and TAD, the expression of miR-20a was detected in the aortic media from TAD patients (n = 10) and donor controls (n = 26). Weigert staining revealed more breakages in the elastic membrane in the aorta media of the TAD group than that in the control group (Fig. 2A). The expression of elastin protein in aortic media tissues from the TAD group was significantly lower than that in the control group (Fig. 2B-C). Conversely, the expression level of miR-20a was dramatically higher in aortic media from the TAD group compared to the control group (Fig. 2D). A significantly negative correlation was found between miR-20a level and elastin expression (correlation coefficient = −0.693, P < 0.01. Fig. 2E). A TAD mouse model was then induced by BAPN and Ang II. A total of 5 mice (25 %) died from TAD rupture (Supplemental Fig. S2). Cardiac echocardiography revealed significant dilatation in the thoracic aorta in the surviving mice from the TAD group (n = 15) (Fig. 2F). The maximum diameter of ascending aorta in the TAD group was significantly larger than that in the control group (1.93 ± 0.25 mm vs. 1.39 ± 0.11 mm, P < 0.01. Fig. 2G). Weigert staining demonstrated significant destruction in the elastic membrane in TAD mice (Fig. 2H). Results of the qRT-PCR assay confirmed that the expression of miR-20a was significantly elevated in thoracic aorta tissues from TAD mice (Fig. 2I). These findings suggested that damage to elastic fibers might be associated with the increased expression of miR-20a.

### 3.3 miR-20a regulated the invasive activity of VSMCs under PDGF-bb stimulation

**Fig. 3:**
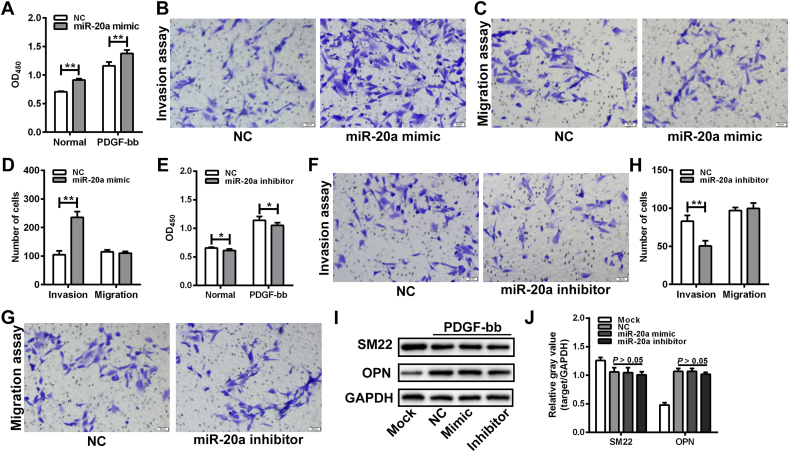
miR-20a regulated the invasive activity of VSMCs under PDGF-bb stimulation. (A) CCK-8 assay of the growth activity of VSMCs transfected with miR-20a mimic under normal or PDGF-bb stimulation conditions. n = 6 for each group. **P < 0.01. (B–D) Matrigel invasion assay and transwell migration assay of the invasive and migratory ability of VSMCs transfected with miR-20a mimic. The number of invaded or migrated VSMCs was calculated under 5 random fields of view. **P < 0.01. (E) CCK-8 assay of the growth activity of VSMCs transfected with miR-20a inhibitor under normal or PDGF-bb stimulation conditions. n = 6 for each group. *P < 0.05. (F–H) Matrigel invasion assay and transwell migration assay of the invasive and migratory ability of VSMCs transfected with miR-20a inhibitor. The number of invaded or migrated VSMCs was calculated under 5 random fields of view. **P < 0.01. (I and J) Western blot assay of SM22α and OPN expression in VSMCs transfected with miR-20a mimic or inhibitor under PDGF-bb stimulation condition. The relative gray values of target proteins were evaluated by Image J software. GAPDH was used as a loading control. n = 3 for each group.

To ascertain the role of miR-20a in the function of aortic VSMCs, a gain-of-function experiment of miR-20a was performed in the isolated VSMCs. Transfection with miR-20a mimic significantly elevated the expression level of miR-20a in VSMCs, while transfection of miR-20a inhibitor significantly reduced the expression level of miR-20a (Supplemental Fig. S3). Compared with the NC group, the growth activity of VSMCs in the miR-20a mimic group was markedly increased under both normal and PDGF-bb stimulation conditions (Fig. 3A). The Matrigel invasion assay showed that the number of invaded VSMCs in the miR-20a mimic group was significantly more than that in the NC group. However, the transwell migration assay showed no significant difference in the number of migrated cells between miR-20a mimic and NC groups (Fig. 3B-D). Following this, a loss-of-function experiment was conducted in VSMCs by transfection of miR-20a inhibitor. Silencing of miR-20a slightly inhibited the growth activity of VSMCs under both normal and PDGF-bb stimulation conditions (Fig. 3E). The number of invaded VSMCs in the miR-20a inhibitor group was significantly less than in the NC group. There was no significant difference in the number of migrated cells between NC and miR-20a inhibitor groups (Fig. 3F-H). Western bolt assay indicated that the protein expression of phenotypic markers (α-SMA, SM22, and OPN) did not change significantly in VSMCs after overexpression or silencing of miR-20a under PDGF-bb stimulation conditions (Fig. 3I-J).

### 3.4 Silencing of miR-20a alleviated the over-activation of MMP2 in VSMCs

**Fig. 4:**
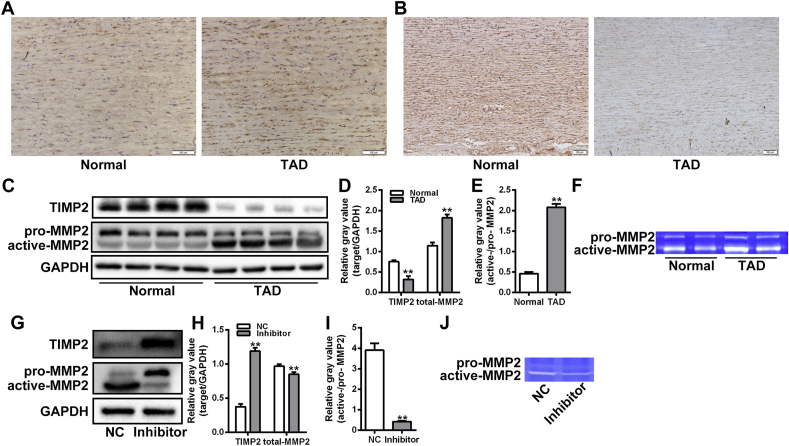
Silencing of miR-20a alleviated the over-activation of MMP2 in VSMCs. (A and B) immunohistochemistry assay of MMP2 and TIMP2 expression in thoracic aorta tissue sections from normal and TAD groups. (C–E) Western blot assay of MMP2 and TIMP2 expression in thoracic aorta tissues from normal (n = 10) and TAD (n = 26) groups. The relative gray values of target proteins were evaluated by Image J software. GAPDH was used as a loading control. **P < 0.01. (F) Gelatin zymography assay of the activity of MMP2 in thoracic aorta tissues from normal and TAD groups. (G–I) Western blot assay of MMP2 and TIMP2 expression in VSMCs transfected with miR-20a inhibitor under PDGF-bb stimulation condition. The relative gray values of target proteins were evaluated by Image J software. GAPDH was used as a loading control. **P < 0.01. (J) Gelatin zymography assay of the activity of MMP2 in VSMCs transfected with miR-20a inhibitor under PDGF-bb stimulation condition.

Imbalance of MMP2/TIMP2 is a critical mediator of VSMC invasion in pathological vascular remodeling. Immunohistochemistry assay showed that the expression level of MMP2 in TAD lesion was significantly higher than that in normal aorta, while the expression of TIMP2 was considerably decreased in TAD lesion (Fig. 4A-B). Western blot assay further demonstrated that the levels of active MMP2 were significantly elevated in the TAD group compared to the control group (Fig. 4C-E). Gelatin zymography assay verified the over-activation of MMP2 in TAD lesions (Fig. 4F). Subsequently, the role of miR-20a in the activation of MMP2 was investigated in VSMCs. Western blot assay showed that the decreased expression of active-MMP2 and increased expression of pro-MMP2 were detected in VSMCs transfected with miR-20a inhibitor under PDGF-bb stimulation, as well as the increased expression of TIMP2 (Fig. 4G-I). Gelatin zymography assay further confirmed that silencing of miR-20a alleviated the overactivation of MMP2 in VSMCs (Fig. 4J).

### 3.5 TIMP2 was a novel target gene of miR-20a in VSMCs

**Fig. 5:**
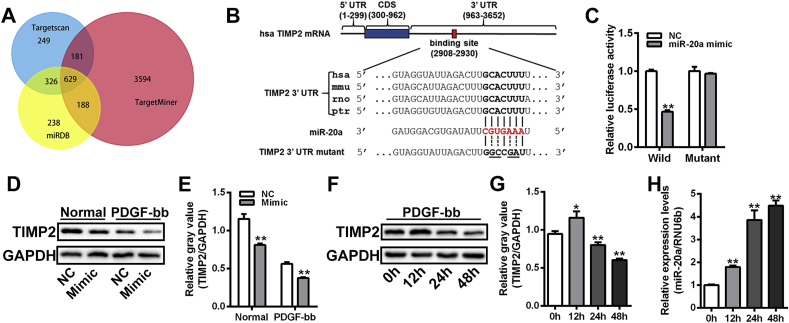
TIMP2 was a novel target gene of miR-20a in VSMCs. (A) Venn diagram of the number of miR-20a target genes predicted by 2 bioinformatics algorithms. (B) A schematic of the full-length information of human TIMP2 mRNA. One 23-nt binding region of miR-20a located in the 3′-UTR of TIMP2. Sequences in bold font are binding sites for the seed sequences of miR-20a (red font). (C) Dual-luciferase reporter assay of the binding between miR-20a and TIMP2 3′ UTR in VSMCs. n = 3 for each group. **P < 0.01. (D and E), Western blot assay of TIMP2 expression in VSMCs transfected with miR-20a mimic under normal and PDGF-bb stimulation conditions. The relative gray values of target proteins were evaluated by Image J software. GAPDH was used as a loading control. n = 3 for each group. **P < 0.01. (F and G) Western blot assay of TIMP2 expression in VSMCs stimulated by PDGF-bb for different times. The relative gray values of target proteins were evaluated by Image J software. GAPDH was used as a loading control. n = 3 for each group. **P < 0.01. (H) qRT-PCR assay of the expression of miR-20a in VSMCs stimulated by PDGF-bb for different times. RNU6b was used as a loading control. n = 3 for each group. **P < 0.01.

To elucidate the mechanism by which miR-20a promoted the overactivation of MMP2 in VSMCs, multiple bioinformatics algorithms were utilized to screen the potential targets of miR-20a. A total of 629 genes, including TIMP2, were predicted as potential targets of miR-20a (Fig. 5A). The RNA-hybrid program displayed one sequence-conserved binding site of miR-20a in the 3′ UTR of TIMP2 (Fig. 5B). Dual-luciferase reporter assay was subsequently employed to confirm the interaction between miR-20a on the 3′ UTR of TIMP2. The activity of the luciferase reporter containing wild-type TIMP2 3’ UTR was significantly reduced in VSMCs transfected with miR-20a mimic, whereas the activity of the mutant reporter was unaffected (Fig. 5C). Western blot analysis confirmed that the protein expression of TIMP2 was significantly decreased in VSMCs transfected with miR-20a mimic under normal and PDGF-bb stimulation conditions (Fig. 5D-E). It was observed that the protein expression of TIMP2 in VSMCs increased significantly after 12-h PDGF-bb stimulation but then decreased after 24 h (Fig. 5F-G). qRT-PCR analysis showed that the expression of miR-20a was significantly upregulated after 12-h PDGF-bb stimulation and remained elevated thereafter (Fig. 5H). These findings suggested that the dynamic changes of TIMP2 expression in VSMCs might be associated with the accumulation of miR-20a.

### 3.6 Enhanced expression of miR-20a is directly driven by Nanog in VSMCs

**Fig. 6:**
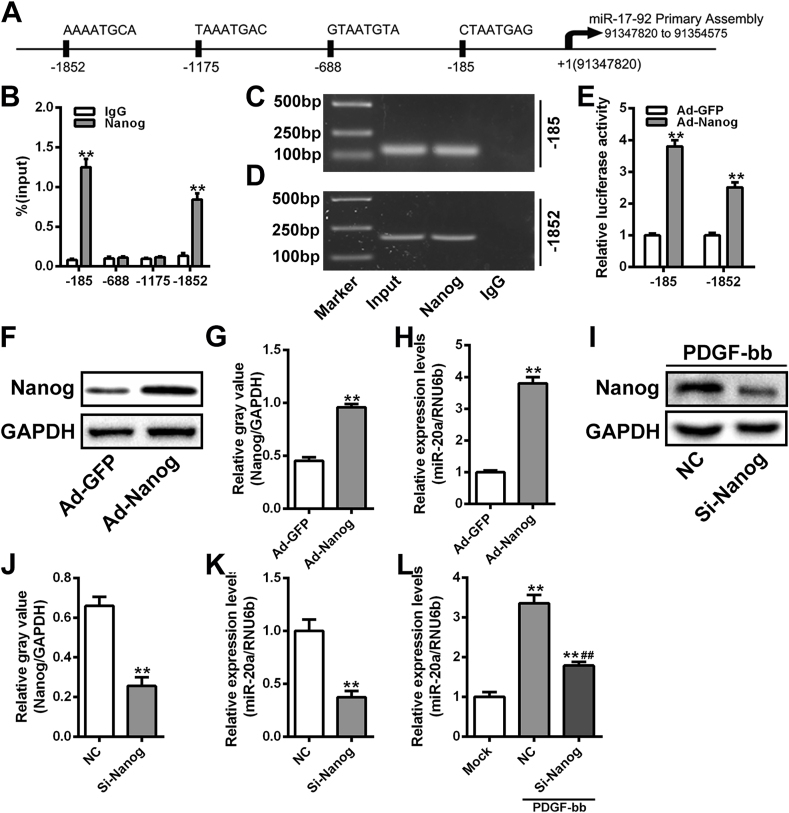
Enhanced expression of miR-20a is directed driven by Nanog in VSMCs. (A) A schematic of the 4 potential binding sites of Nanog in the promoter region of miR-17-92 cluster. (B) ChIP-PCR assay of Nanog directly binding with potential binding sites in the promoter region of miR-17-92 cluster. **P < 0.01. (C and D) Agarose gel electrophoresis assay of the ChIP-PCR amplified products. (E) Dual-luciferase reporter assay of the binding between Nanog and the promoter region of miR-17-92 cluster in VSMCs. n = 3 for each group. **P < 0.01. (F and G) Western blot assay of Nanog expression in VSMCs transfected with Ad-Nanog. The relative gray values of target proteins were evaluated by Image J software. GAPDH was used as a loading control. n = 3 for each group. **P < 0.01. (H) qRT-PCR assay of the expression of miR-20a in VSMCs transfected with Ad-Nanog. RNU6b was used as a loading control. n = 3 for each group. **P < 0.01. (I and J) Western blot assay of Nanog expression in VSMCs transfected with si-Nanog. The relative gray values of target proteins were evaluated by Image J software. GAPDH was used as a loading control. n = 3 for each group. **P < 0.01. (K) qRT-PCR assay of the expression of miR-20a in VSMCs transfected with si-Nanog. RNU6b was used as a loading control. n = 3 for each group. **P < 0.01. (L) qRT-PCR assay of the expression of miR-20a in Nanog-silencing VSMCs under PDGF-bb stimulation condition. RNU6b was used as a loading control. n = 3 for each group. **P < 0.01.

It has been reported that the transcription of miR-17-92 cluster was driven by Nanog in neural stem cells [29], and a Nanog-driven Foxm1-miRNA network regulates the self-renewal capacity of neural stem cells [30]. Our previous study demonstrated that Nanog was highly expressed in TAD aortic wall and in synthetic VSMCs [19]. Consequently, we explored the role and mechanism of Nanog in regulating miR-20a expression in VSMCs. Bioinformatics analysis using the JASPAR database revealed four potential Nanog binding sites in the promoter region of the miR-17-92 cluster (Fig. 6A). Primer sets flanking these predicted regions were used to amplify two distinct areas, located 185 bp and 1852 bp upstream of the transcription start site of the miR-17-92 cluster, from DNA immunoprecipitated with a Nanog antibody (Fig. 6B-D), suggesting Nanog may directly bind to the promoter of miR-17-92 cluster in VSMCs. Dual-luciferase reporter assay confirmed that the activity of the luciferase reporter containing these two regions was significantly increased in VSMCs transfected with Ad-Nanog (Fig. 6E), verifying these regions as functional transcription binding sites for Nanog. Subsequently, the effect of Nanog on miR-20a expression in VSMCs was investigated. Compared to the Ad-GFP group, the expression of miR-20a was increased in VSMCs of the Ad-Nanog group (Fig. 6F-H). Conversely, the expression of miR-20a in VSMCs from the si-Nanog group was significantly lower than that from the NC group (Fig. 6I-K). Notably, VSMCs with Nanog silenced showed a diminished response to the upregulation of miR-20a induced by PDGF-bb.

## 4 Discussion

The present study found increased expression of the miR-17-92 cluster in thoracic aorta tissues from TAD patients and TAD mice, as well as a significant negative correlation between miR-20a and elastin expression in TAD aortas. The study also elucidated the role and molecular mechanism of miR-20a in the imbalance of MMP2/TIMP2 in VSMCs. The enhanced expression of miR-20a, which was directly driven by Nanog, exacerbated the overactivation of MMP2 in VSMCs by suppressing the expression of TIMP2. The results highlight the critical role of miR-20a in maintaining aortic wall homeostasis during the pathogenesis of TAD.

TAD is a deadly medical emergency with very high mortality, especially Stanford type A TAD, the mortality of which was 1–2% per hour in the first 24 h and almost 50 % within the first week [31]. Surgical repair is an effective treatment for type A TAD, yet the postoperative mortality remains high, ranging from about 4.7 % to 18.4 % [32,33]. Previous studies have shown that excessive ECM degradation in the aorta plays a crucial role in the pathophysiologic process of TAD. Our study also identified increased breaks in elastin fibers and reduced elastin expression in the TAD aorta. ECM degradation leads to the destruction of elastic fibers, increased collagen deposition, and a reduction in aortic medial thickness, all of which were risk factors for TAD [18,34].

MMP2 is an essential ECM protease with broad functions in ECM remodeling. It belongs to the family of zinc-dependent endopeptidases, most of which exist in an inactive form that includes an inhibitory peptide masking the zinc-binding domain [35]. To become active, the inhibitory peptide must be removed by other MMPs, plasmin, or neutrophil elastase [36]. MMP2 is one of the most extensively studied MMP members, and its overactivation is closely associated with the development of TAD [37]. Numerous studies have demonstrated increased levels of active-MMP2 in the aortic wall of aortic aneurysm or dissection [38,39]. In this study, MMP2 was found to be significantly overactivated in the TAD aorta, a finding consistent with previous research and indicative of MMP2's critical role in TAD.

The present study confirmed a significantly decreased in the expression of TIMP2 protein in the TAD aorta. TIMP2, a secreted protein with 194 amino acids, functions to inhibit the activation of MMP2 [40]. The inhibitory function of TIMP2 relies on its conserved “wedge-shaped” N-terminal region. When TIMP2 combines with MMP2, this “wedge-shaped” is embedded in the functional area of MMP2, thereby inhibiting its activity [41]. Several studies have reported decreased expression of TIMP2 in the aorta from TAD and aortic aneurysm patients [42,43]. Additionally, it has been reported that overexpression of TIMP2 can protect the aorta from dilation in rats [44], suggesting TIMP2 could be a potential therapeutic target for aortic diseases. In this study, the expression of TIMP2 in VSMCs was found to be regulated by miR-20a. Silencing of miR-20a could alleviate the overactivation of MMP2 in VSMCs by enhancing TIMP2 expression. These findings broaden the potential interventional approaches to targeting TIMP2 for therapeutic purposes.

Our previous study showed that Nanog was highly expressed in the TAD aorta and could induce the upregulation of MMP2 [19]. The present study further found that Nanog promoted the imbalance between MMP2 and TIMP2 in VSMCs by driving the expression of miR-20a. It has been reported that the TIMP-MMP balance in aorta tissues may be controlled of the miR-17-92 cluster in patients with BAV [45]. Significant repression of the miR-17-92 cluster was observed in grafted veins in response to arterial cyclic stretch, and increasing the level of miR-20a in venous smooth muscle cells could attenuate neointimal hyperplasia in grafted vessels [46]. These results suggest that interruption of the Nonog-miR-20a axis might be a potential strategy for the prevention and treatment of TAD.

## 5 Conclusion

Several limitations of this study should be noted. First, the in-vitro experiment could not fully mimic the pathological aortic remodeling during TAD pathogenesis. It is essential to confirm the roles and mechanisms of Nanog/miR-20a/TIMP2 using an in-vivo TAD model, specifically one involving VSMC-specific conditional miR-20a knockout mice. Second, the therapeutic efficacy of targeting miR-20a to alleviate the TAD progression has not yet been evaluated. Emerging targeted drug delivery systems, particularly those based on biomimetic nanoparticles, may prove useful for lesion-site specific administration of miR-20a inhibitors. In summary, the present study has provided new insights into the mechanisms underlying TAD pathogenesis, and gave the evidence that Nanog-miR-20a axis could be a promising therapeutic target for TAD.

## Ethics approval and consent to participate

The study conformed to the principles outlined in the Declaration of Helsinki and was approved by the Medical Ethics Committee in Changhai Hospital. Written informed consent was obtained from all the participants before enrollment.

## Funding

This work was supported by the 10.13039/501100001809National Natural Science Foundation of China (81900421), Shanghai Science and Technology Innovation Action Plan “Science and Technology Support Project in Biomedical Science” (21S11906000), and the Incubation Project of 10.13039/501100017536Shanghai Pulmonary Hospital (fkzr2109).

## CRediT authorship contribution statement

**Zhao An:** Writing – original draft, Investigation, Funding acquisition, Conceptualization. **Yangyong Sun:** Writing – review & editing, Investigation. **Xiaodong Yang:** Data curation. **Jingwen Zhou:** Validation. **Yongchao Yu:** Validation. **Boyao Zhang:** Validation. **Zhiyun Xu:** Conceptualization. **Yuming Zhu:** Conceptualization. **Guokun Wang:** Writing – review & editing, Writing – original draft, Methodology, Funding acquisition, Conceptualization.

## Declaration of competing interest

The authors declare that they have no known competing financial interests or personal relationships that could have appeared to influence the work reported in this paper.

☐The authors declare the following financial interests/personal relationships which may be considered as potential competing interests:
