# Supplementary material for: Enhanced expression of miR-20a driven by nanog exacerbated the degradation of extracellular matrix in thoracic aortic dissection
Source: Noncoding RNA Res. 2024 May 20;9(4):1040–9. doi: 10.1016/j.ncrna.2024.05.006 (PMC11254500; doi:10.1016/j.ncrna.2024.05.006)
Supplement: Multimedia component 1 [file mmc1.docx]

**Supplemental Figures**


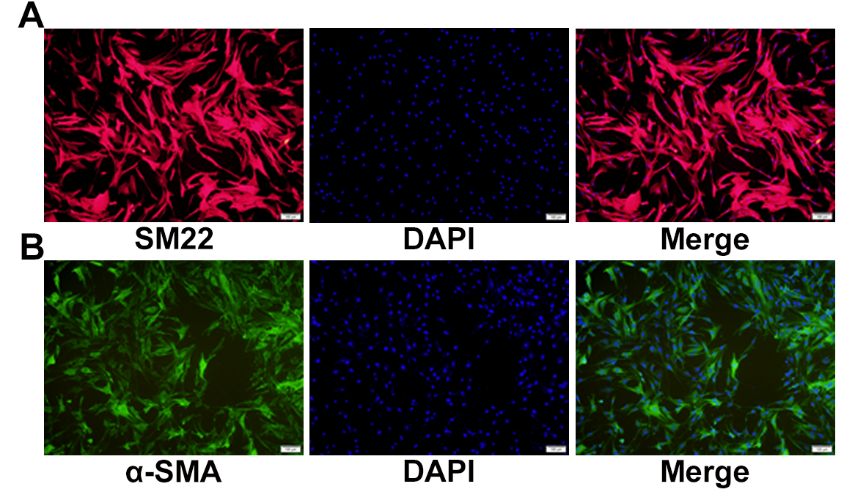


Figure S1 Identification of primary isolated VSMCs by immunofluorescence of SM22 (A) and α-SMA (B). Nucleus was stained with DAPI (blue). Scalar bar = 100 μm.


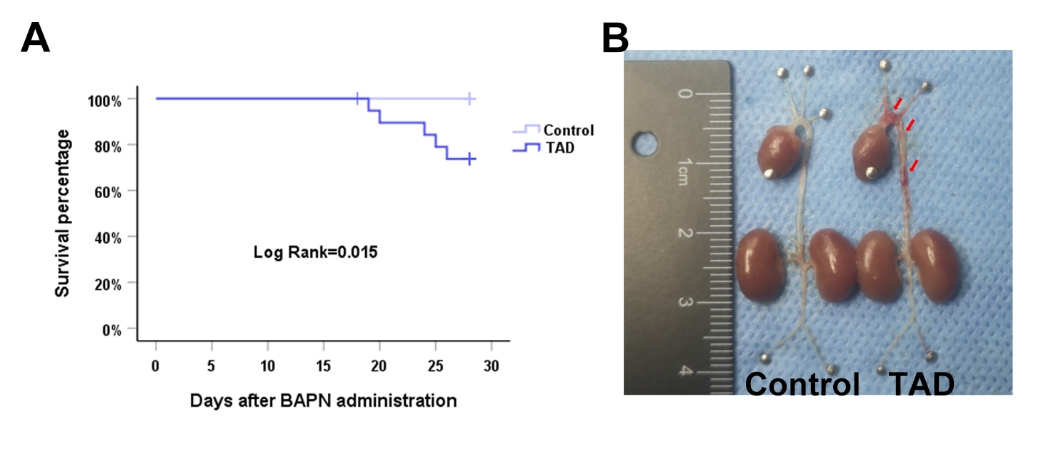


Figure S2 Establishment of TAD mouse model. A, Survival analysis during modelling of TAD mice. n=20 for each group. B, Dissections (red arrows) in aortic wall in aorta specimen from TAD group.


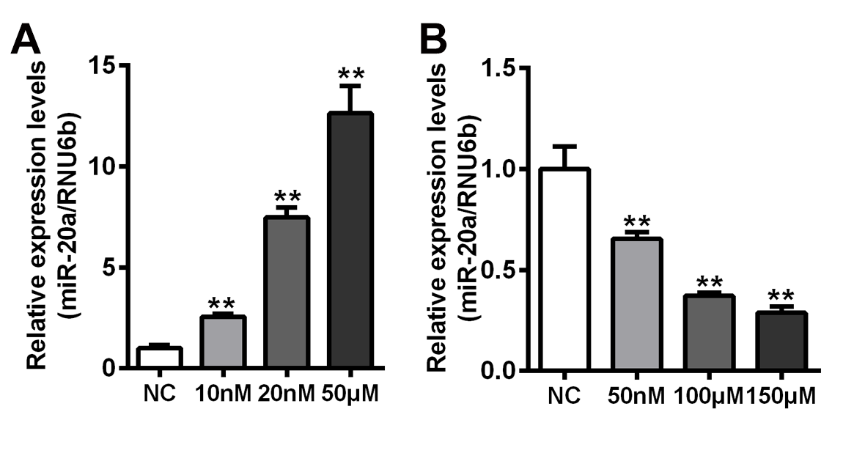


Figure S3 qRT-PCR assay of the expression of miR-20a in VSMCs transfected with miR-20a mimic (A) or miR-20a inhibitor (B) of different concentrations. RNU6b was used as a loading control. n=3 for each group. **P<0.01.
