# Supplementary material for: Enhanced expression of miR-20a driven by nanog exacerbated the degradation of extracellular matrix in thoracic aortic dissection
Source: Noncoding RNA Res. 2024 May 20;9(4):1040–9. doi: 10.1016/j.ncrna.2024.05.006 (PMC11254500; doi:10.1016/j.ncrna.2024.05.006)
Supplement: Multimedia component 2 [file mmc2.docx]

**Supplemental Tables**

**Table S1 The sequences of primers used in the PCR assay**

| **Name** | **Sequences (5’-3’)** |
| --- | --- |
| miR-17 | stem-loop: CTCAACTGGTGTCGTGGAGTCGGCAATTCAGTTGAGCTACCTGC  forward: ACACTCCAGCTGGGCAAAGTGCTTACAGTGC; reverse: CTCAACTGGTGTCGTGGAGT |
| miR-18a | stem-loop: CTCAACTGGTGTCGTGGAGTCGGCAATTCAGTTGAGCTATCTGC  forward: ACACTCCAGCTGGGTAAGGTGCATCTAGTGC; reverse: CTCAACTGGTGTCGTGGAGT |
| miR-19a | stem-loop: CTCAACTGGTGTCGTGGAGTCGGCAATTCAGTTGAGTCAGTTTT  forward: ACACTCCAGCTGGGTGTGCAAATCTATGCAA; reverse: CTCAACTGGTGTCGTGGAGT |
| miR-19b | stem-loop: CTCAACTGGTGTCGTGGAGTCGGCAATTCAGTTGAGTCAGTTTT  forward: ACACTCCAGCTGGGTGTGCAAATCCATGCAA; reverse: CTCAACTGGTGTCGTGGAGT |
| miR-20a | stem-loop: CTCAACTGGTGTCGTGGAGTCGGCAATTCAGTTGAGCTACCTGC  forward: ACACTCCAGCTGGGTAAAGTGCTTATAGTGC; reverse: CTCAACTGGTGTCGTGGAGT |
| miR-92a | stem-loop: CTCAACTGGTGTCGTGGAGTCGGCAATTCAGTTGAGACAGGCCG  forward: ACACTCCAGCTGGGTATTGCACTTGTCCCG; reverse: CTCAACTGGTGTCGTGGAGT |
| RNU6b | stem-loop: GTCGTATCCAGTGCAGGGTCCGAGGTATTCGCACTGGATACGACAAAATATGGAAC  forward: GCCCCTGCGCAAGGATGAC; reverse: GTGCAGGGTCCGAGGT |
| GAPDH | forward: ACCACCATGGAGAAGGCTG; reverse: GGTCATGAGTCCTTCCACGA |

**Table S2 The detailed information of antibodies**

| Name | Description | Company | Application information |
| --- | --- | --- | --- |
| anti-α-SMA | Mouse monoclonal | Boster, Wuhan, China | 1: 1000 dilution for WB; 1:200 dilution for IF |
| anti-OPN | Rabbit monoclonal | Abcam, Cambridge, UK | 1:1000 dilution in WB; 1:50 dilution for IF |
| anti-elastin | Rabbit monoclonal | Abcam, Cambridge, UK | 1:1000 dilution in WB |
| anti-SM22α | Rabbit polyclonal | Abcam, Cambridge, UK | 1:1000 dilution in WB; 1:200 dilution for IF |
| anti-TIMP2 | Rabbit polyclonal | Abcam, Cambridge, UK | 1:1000 dilution in WB; 1:200 dilution in IHC |
| anti-MMP2 | Rabbit polyclonal | Santa Cruz Biotechnology, Santa Cruz, CA, USA | 1:1000 dilution in WB; 1:200 dilution in IHC |
| anti-GAPDH | Mouse monoclonal | Proteintech, Wuhan, China | 1:5000 dilution for WB |

**Table S3 The detailed information of synthetic fragments**

| Name | Sequences (5’-3’) | Transfection concentration |
| --- | --- | --- |
| miR-20a mimic | UAAAGUGCUUAUAGUGCAGGUAG | 20 nmol/L |
| miR-20a inhibitor | CUACCUGCACUAUAAGCACUUUA | 100 nmol/L |

**Table S4 The potential target genes of miR-20a predicted by multiple algorithms**

| **No.** | **Gene Symbol** | **Gene Description** | **No.** | **Gene Symbol** | **Gene Description** |
| --- | --- | --- | --- | --- | --- |
| 1 | ANKRD52 | ankyrin repeat domain 52 | 29 | USP46 | ubiquitin specific peptidase 46 |
| 2 | DYNC1LI2 | dynein cytoplasmic 1 light intermediate chain 2 | 30 | VLDLR | very low density lipoprotein receptor |
| 3 | NAPEPLD | N-acyl phosphatidylethanolamine phospholipase D | 31 | ZNF800 | zinc finger protein 800 |
| 4 | PKD2 | polycystin 2, transient receptor potential cation channel | 32 | ZNF827 | zinc finger protein 827 |
| 5 | ZFYVE26 | zinc finger FYVE-type containing 26 | 33 | AKTIP | AKT interacting protein |
| 6 | ARHGAP12 | Rho GTPase activating protein 12 | 34 | ANKIB1 | ankyrin repeat and IBR domain containing 1 |
| 7 | ARID4B | AT-rich interaction domain 4B | 35 | EPHA4 | EPH receptor A4 |
| 8 | BRMS1L | BRMS1 like transcriptional repressor | 36 | EPHA5 | EPH receptor A5 |
| 9 | CFL2 | cofilin 2 | 37 | FBXL5 | F-box and leucine rich repeat protein 5 |
| 10 | CLOCK | clock circadian regulator | 38 | FCHO2 | FCH domain only 2 |
| 11 | EZH1 | enhancer of zeste 1 polycomb repressive complex 2 subunit | 39 | FRMD6 | FERM domain containing 6 |
| 12 | GPR6 | G protein-coupled receptor 6 | 40 | HSPA8 | heat shock protein family A (Hsp70) member 8 |
| 13 | ITGB8 | integrin subunit beta 8 | 41 | KIAA0513 | KIAA0513 |
| 14 | ITPRIPL2 | ITPRIP like 2 | 42 | KLHL28 | kelch like family member 28 |
| 15 | KCNK10 | potassium two pore domain channel subfamily K member 10 | 43 | MKRN1 | makorin ring finger protein 1 |
| 16 | MAP3K2 | mitogen-activated protein kinase kinase kinase 2 | 44 | NCOA3 | nuclear receptor coactivator 3 |
| 17 | NPAT | nuclear protein, coactivator of histone transcription | 45 | NFAT5 | nuclear factor of activated T cells 5 |
| 18 | PLEKHA3 | pleckstrin homology domain containing A3 | 46 | RGL1 | ral guanine nucleotide dissociation stimulator like 1 |
| 19 | PTPN4 | protein tyrosine phosphatase, non-receptor type 4 | 47 | RNF128 | ring finger protein 128, E3 ubiquitin protein ligase |
| 20 | RAB22A | RAB22A, member RAS oncogene family | 48 | RPS6KA5 | ribosomal protein S6 kinase A5 |
| 21 | REEP3 | receptor accessory protein 3 | 49 | VANGL1 | VANGL planar cell polarity protein 1 |
| 22 | RRAGD | Ras related GTP binding D | 50 | ZBTB4 | zinc finger and BTB domain containing 4 |
| 23 | SACS | sacsin molecular chaperone | 51 | ZNF512B | zinc finger protein 512B |
| 24 | SAR1B | secretion associated Ras related GTPase 1B | 52 | ATG16L1 | autophagy related 16 like 1 |
| 25 | STK17B | serine/threonine kinase 17b | 53 | BCL11B | BCL11B, BAF complex component |
| 26 | TBC1D20 | TBC1 domain family member 20 | 54 | BNIP2 | BCL2 interacting protein 2 |
| 27 | TBC1D9 | TBC1 domain family member 9 | 55 | CCND1 | cyclin D1 |
| 28 | TXNIP | thioredoxin interacting protein | 56 | EFCAB14 | EF-hand calcium binding domain 14 |
| **No.** | **Gene Symbol** | **Gene Description** | **No.** | **Gene Symbol** | **Gene Description** |
| 57 | EIF5A2 | eukaryotic translation initiation factor 5A2 | 86 | SERTAD2 | SERTA domain containing 2 |
| 58 | FOXJ3 | forkhead box J3 | 87 | SPRED1 | sprouty related EVH1 domain containing 1 |
| 59 | FRS2 | fibroblast growth factor receptor substrate 2 | 88 | STXBP5 | syntaxin binding protein 5 |
| 60 | GLIS3 | GLIS family zinc finger 3 | 89 | TGFBR2 | transforming growth factor beta receptor 2 |
| 61 | GPR63 | G protein-coupled receptor 63 | 90 | TNFRSF21 | TNF receptor superfamily member 21 |
| 62 | ITGA4 | integrin subunit alpha 4 | 91 | TRIM36 | tripartite motif containing 36 |
| 63 | KMT2B | lysine methyltransferase 2B | 92 | USP31 | ubiquitin specific peptidase 31 |
| 64 | MYT1L | myelin transcription factor 1 like | 93 | VSX1 | visual system homeobox 1 |
| 65 | NABP1 | nucleic acid binding protein 1 | 94 | ZBTB41 | zinc finger and BTB domain containing 41 |
| 66 | NR2C2 | nuclear receptor subfamily 2 group C member 2 | 95 | ZFP91 | ZFP91 zinc finger protein |
| 67 | TRIP10 | thyroid hormone receptor interactor 10 | 96 | ZNF652 | zinc finger protein 652 |
| 68 | USP3 | ubiquitin specific peptidase 3 | 97 | ZNF704 | zinc finger protein 704 |
| 69 | VASH2 | vasohibin 2 | 98 | ARMC8 | armadillo repeat containing 8 |
| 70 | ZFYVE9 | zinc finger FYVE-type containing 9 | 99 | B3GALT2 | beta-1,3-galactosyltransferase 2 |
| 71 | ANKRD29 | ankyrin repeat domain 29 | 100 | CEP97 | centrosomal protein 97 |
| 72 | ARID4A | AT-rich interaction domain 4A | 101 | E2F5 | E2F transcription factor 5 |
| 73 | ATAD2 | ATPase family, AAA domain containing 2 | 102 | EPHA7 | EPH receptor A7 |
| 74 | CHRM2 | cholinergic receptor muscarinic 2 | 103 | F3 | coagulation factor III, tissue factor |
| 75 | DPYSL5 | dihydropyrimidinase like 5 | 104 | HAS2 | hyaluronan synthase 2 |
| 76 | EGLN3 | egl-9 family hypoxia inducible factor 3 | 105 | IRF9 | interferon regulatory factor 9 |
| 77 | FAM117B | family with sequence similarity 117 member B | 106 | KAT2B | lysine acetyltransferase 2B |
| 78 | FGD5 | FYVE, RhoGEF and PH domain containing 5 | 107 | LAMA3 | laminin subunit alpha 3 |
| 79 | FJX1 | four-jointed box kinase 1 | 108 | LIMK1 | LIM domain kinase 1 |
| 80 | LAPTM4A | lysosomal protein transmembrane 4 alpha | 109 | LPGAT1 | lysophosphatidylglycerol acyltransferase 1 |
| 81 | PEX5L | peroxisomal biogenesis factor 5 like | 110 | MASTL | microtubule associated serine/threonine kinase like |
| 82 | PHC3 | polyhomeotic homolog 3 | 111 | NAGK | N-acetylglucosamine kinase |
| 83 | PRRG1 | proline rich and Gla domain 1 | 112 | NCKAP5 | NCK associated protein 5 |
| 84 | PURB | purine rich element binding protein B | 113 | PFKP | phosphofructokinase, platelet |
| 85 | RBL2 | RB transcriptional corepressor like 2 | 114 | PGM2L1 | phosphoglucomutase 2 like 1 |
| **No.** | **Gene Symbol** | **Gene Description** | **No.** | **Gene Symbol** | **Gene Description** |
| 115 | PPP6C | protein phosphatase 6 catalytic subunit | 144 | PTPRD | protein tyrosine phosphatase, receptor type D |
| 116 | PTHLH | parathyroid hormone like hormone | 145 | RAB5B | RAB5B, member RAS oncogene family |
| 117 | RASL11B | RAS like family 11 member B | 146 | SNTB2 | syntrophin beta 2 |
| 118 | SALL1 | spalt like transcription factor 1 | 147 | TMEM127 | transmembrane protein 127 |
| 119 | SLC17A7 | solute carrier family 17 member 7 | 148 | TOPORS | TOP1 binding arginine/serine rich protein |
| 120 | SPOPL | speckle type BTB/POZ protein like | 149 | UBE2Q2 | ubiquitin conjugating enzyme E2 Q2 |
| 121 | UEVLD | UEV and lactate/malate dehyrogenase domains | 150 | XRN1 | 5'-3' exoribonuclease 1 |
| 122 | ABCA1 | ATP binding cassette subfamily A member 1 | 151 | ZBTB20 | zinc finger and BTB domain containing 20 |
| 123 | AGO1 | argonaute RISC catalytic component 1 | 152 | ANO6 | anoctamin 6 |
| 124 | ANKRD17 | ankyrin repeat domain 17 | 153 | ARHGAP26 | Rho GTPase activating protein 26 |
| 125 | BRWD1 | bromodomain and WD repeat domain containing 1 | 154 | BMPR2 | bone morphogenetic protein receptor type 2 |
| 126 | BTBD10 | BTB domain containing 10 | 155 | BNC2 | basonuclin 2 |
| 127 | BTG3 | BTG anti-proliferation factor 3 | 156 | CALD1 | caldesmon 1 |
| 128 | CAPRIN2 | caprin family member 2 | 157 | CCNG2 | cyclin G2 |
| 129 | DDHD1 | DDHD domain containing 1 | 158 | CROT | carnitine O-octanoyltransferase |
| 130 | FAT2 | FAT atypical cadherin 2 | 159 | DENND5B | DENN domain containing 5B |
| 131 | FBXO48 | F-box protein 48 | 160 | DRD1 | dopamine receptor D1 |
| 132 | FZD3 | frizzled class receptor 3 | 161 | ELK3 | ELK3, ETS transcription factor |
| 133 | GAB1 | GRB2 associated binding protein 1 | 162 | IQSEC2 | IQ motif and Sec7 domain 2 |
| 134 | GPR137B | G protein-coupled receptor 137B | 163 | NEDD4L | NEDD4 like E3 ubiquitin protein ligase |
| 135 | HBP1 | HMG-box transcription factor 1 | 164 | PPP1R15B | protein phosphatase 1 regulatory subunit 15B |
| 136 | KIF3B | kinesin family member 3B | 165 | RAP2C | RAP2C, member of RAS oncogene family |
| 137 | KLHL2 | kelch like family member 2 | 166 | SCN1A | sodium voltage-gated channel alpha subunit 1 |
| 138 | KMT2A | lysine methyltransferase 2A | 167 | SEMA4B | semaphorin 4B |
| 139 | LDLRAP1 | low density lipoprotein receptor adaptor protein 1 | 168 | SLITRK3 | SLIT and NTRK like family member 3 |
| 140 | LIMA1 | LIM domain and actin binding 1 | 169 | STRIP2 | striatin interacting protein 2 |
| 141 | NIN | ninein | 170 | TAOK3 | TAO kinase 3 |
| 142 | PDE3B | phosphodiesterase 3B | 171 | ULK1 | unc-51 like autophagy activating kinase 1 |
| 143 | PTPN3 | protein tyrosine phosphatase, non-receptor type 3 | 172 | YOD1 | YOD1 deubiquitinase |
| **No.** | **Gene Symbol** | **Gene Description** | **No.** | **Gene Symbol** | **Gene Description** |
| 173 | ACSL4 | acyl-CoA synthetase long chain family member 4 | 202 | FEM1C | fem-1 homolog C |
| 174 | AP2B1 | adaptor related protein complex 2 subunit beta 1 | 203 | FNDC3B | fibronectin type III domain containing 3B |
| 175 | C14orf28 | chromosome 14 open reading frame 28 | 204 | IL6ST | interleukin 6 signal transducer |
| 176 | CEP120 | centrosomal protein 120 | 205 | KLF11 | Kruppel like factor 11 |
| 177 | DNAJC16 | DnaJ heat shock protein family (Hsp40) member C16 | 206 | LDLR | low density lipoprotein receptor |
| 178 | EGR2 | early growth response 2 | 207 | LYST | lysosomal trafficking regulator |
| 179 | ETV1 | ETS variant 1 | 208 | NBEA | neurobeachin |
| 180 | HECTD2 | HECT domain E3 ubiquitin protein ligase 2 | 209 | PCDHA1 | protocadherin alpha 1 |
| 181 | ISM2 | isthmin 2 | 210 | PCDHA10 | protocadherin alpha 10 |
| 182 | L3MBTL3 | L3MBTL3, histone methyl-lysine binding protein | 211 | PCDHA11 | protocadherin alpha 11 |
| 183 | LASP1 | LIM and SH3 protein 1 | 212 | PCDHA12 | protocadherin alpha 12 |
| 184 | LRCH1 | leucine rich repeats and calponin homology domain containing 1 | 213 | PCDHA13 | protocadherin alpha 13 |
| 185 | LRP8 | LDL receptor related protein 8 | 214 | PCDHA2 | protocadherin alpha 2 |
| 186 | NTN4 | netrin 4 | 215 | PCDHA3 | protocadherin alpha 3 |
| 187 | OCRL | OCRL, inositol polyphosphate-5-phosphatase | 216 | PCDHA4 | protocadherin alpha 4 |
| 188 | RAB11FIP1 | RAB11 family interacting protein 1 | 217 | PCDHA5 | protocadherin alpha 5 |
| 189 | RACGAP1 | Rac GTPase activating protein 1 | 218 | PCDHA6 | protocadherin alpha 6 |
| 190 | RGMB | repulsive guidance molecule BMP co-receptor b | 219 | PCDHA7 | protocadherin alpha 7 |
| 191 | SUCO | SUN domain containing ossification factor | 220 | PCDHA8 | protocadherin alpha 8 |
| 192 | TMEM168 | transmembrane protein 168 | 221 | PCDHAC1 | protocadherin alpha subfamily C, 1 |
| 193 | TMEM64 | transmembrane protein 64 | 222 | PCDHAC2 | protocadherin alpha subfamily C, 2 |
| 194 | TNKS2 | tankyrase 2 | 223 | PRR15 | proline rich 15 |
| 195 | URI1 | URI1, prefoldin like chaperone | 224 | RAB8B | RAB8B, member RAS oncogene family |
| 196 | ANKFY1 | ankyrin repeat and FYVE domain containing 1 | 225 | RAPH1 | Ras association and pleckstrin homology domains 1 |
| 197 | APCDD1 | APC down-regulated 1 | 226 | RB1CC1 | RB1 inducible coiled-coil 1 |
| 198 | CC2D1A | coiled-coil and C2 domain containing 1A | 227 | SLC16A9 | solute carrier family 16 member 9 |
| 199 | CCDC71L | coiled-coil domain containing 71 like | 228 | SMOC1 | SPARC related modular calcium binding 1 |
| 200 | DAB2 | DAB2, clathrin adaptor protein | 229 | TET1 | tet methylcytosine dioxygenase 1 |
| 201 | DERL2 | derlin 2 | 230 | THRA | thyroid hormone receptor alpha |
| **No.** | **Gene Symbol** | **Gene Description** | **No.** | **Gene Symbol** | **Gene Description** |
| 231 | TIAM1 | T cell lymphoma invasion and metastasis 1 | 260 | IL1RAP | interleukin 1 receptor accessory protein |
| 232 | UBE3C | ubiquitin protein ligase E3C | 261 | LHX6 | LIM homeobox 6 |
| 233 | WDFY3 | WD repeat and FYVE domain containing 3 | 262 | MAP3K9 | mitogen-activated protein kinase kinase kinase 9 |
| 234 | ZBTB33 | zinc finger and BTB domain containing 33 | 263 | MCL1 | MCL1, BCL2 family apoptosis regulator |
| 235 | ZFAND4 | zinc finger AN1-type containing 4 | 264 | MFAP3L | microfibril associated protein 3 like |
| 236 | ARHGEF10 | Rho guanine nucleotide exchange factor 10 | 265 | PBX3 | PBX homeobox 3 |
| 237 | CREB1 | cAMP responsive element binding protein 1 | 266 | PITPNA | phosphatidylinositol transfer protein alpha |
| 238 | CSRNP3 | cysteine and serine rich nuclear protein 3 | 267 | PLAGL2 | PLAG1 like zinc finger 2 |
| 239 | DPYSL2 | dihydropyrimidinase like 2 | 268 | ROCK2 | Rho associated coiled-coil containing protein kinase 2 |
| 240 | GOLGA1 | golgin A1 | 269 | SLC22A23 | solute carrier family 22 member 23 |
| 241 | KIF23 | kinesin family member 23 | 270 | SMOC2 | SPARC related modular calcium binding 2 |
| 242 | MAPK1 | mitogen-activated protein kinase 1 | 271 | SRGAP1 | SLIT-ROBO Rho GTPase activating protein 1 |
| 243 | MEX3D | mex-3 RNA binding family member D | 272 | TANC1 | tetratricopeptide repeat, ankyrin repeat and coiled-coil containing 1 |
| 244 | MTMR3 | myotubularin related protein 3 | 273 | TNFAIP1 | TNF alpha induced protein 1 |
| 245 | NAA30 | N(alpha)-acetyltransferase 30, NatC catalytic subunit | 274 | TNKS1BP1 | tankyrase 1 binding protein 1 |
| 246 | OXR1 | oxidation resistance 1 | 275 | USP32 | ubiquitin specific peptidase 32 |
| 247 | S1PR1 | sphingosine-1-phosphate receptor 1 | 276 | WNK3 | WNK lysine deficient protein kinase 3 |
| 248 | SLC16A6 | solute carrier family 16 member 6 | 277 | ZBTB18 | zinc finger and BTB domain containing 18 |
| 249 | SSX2IP | SSX family member 2 interacting protein | 278 | CAPN15 | calpain 15 |
| 250 | WDR37 | WD repeat domain 37 | 279 | DOCK4 | dedicator of cytokinesis 4 |
| 251 | AGFG2 | ArfGAP with FG repeats 2 | 280 | FAT4 | FAT atypical cadherin 4 |
| 252 | APP | amyloid beta precursor protein | 281 | FBXL3 | F-box and leucine rich repeat protein 3 |
| 253 | BTBD7 | BTB domain containing 7 | 282 | FNBP1L | formin binding protein 1 like |
| 254 | CRYBG3 | crystallin beta-gamma domain containing 3 | 283 | FOXK2 | forkhead box K2 |
| 255 | DCBLD2 | discoidin, CUB and LCCL domain containing 2 | 284 | MFN2 | mitofusin 2 |
| 256 | DNAL1 | dynein axonemal light chain 1 | 285 | NUP35 | nucleoporin 35 |
| 257 | FAM13A | family with sequence similarity 13 member A | 286 | PIK3R1 | phosphoinositide-3-kinase regulatory subunit 1 |
| 258 | FBXO21 | F-box protein 21 | 287 | REPS2 | RALBP1 associated Eps domain containing 2 |
| 259 | GOSR1 | golgi SNAP receptor complex member 1 | 288 | SFMBT1 | Scm like with four mbt domains 1 |
| **No.** | **Gene Symbol** | **Gene Description** | **No.** | **Gene Symbol** | **Gene Description** |
| 289 | SMAD5 | SMAD family member 5 | 318 | MAP3K8 | mitogen-activated protein kinase kinase kinase 8 |
| 290 | ZNF148 | zinc finger protein 148 | 319 | MINK1 | misshapen like kinase 1 |
| 291 | AKAP11 | A-kinase anchoring protein 11 | 320 | MYLIP | myosin regulatory light chain interacting protein |
| 292 | ANKRD13C | ankyrin repeat domain 13C | 321 | NANOS1 | nanos C2HC-type zinc finger 1 |
| 293 | ANKRD50 | ankyrin repeat domain 50 | 322 | NTNG1 | netrin G1 |
| 294 | BAHD1 | bromo adjacent homology domain containing 1 | 323 | PLXNA1 | plexin A1 |
| 295 | HIF1A | hypoxia inducible factor 1 subunit alpha | 324 | RAB10 | RAB10, member RAS oncogene family |
| 296 | MIDN | midnolin | 325 | RBBP7 | RB binding protein 7, chromatin remodeling factor |
| 297 | MMP24 | matrix metallopeptidase 24 | 326 | REV3L | REV3 like, DNA directed polymerase zeta catalytic subunit |
| 298 | PDGFRA | platelet derived growth factor receptor alpha | 327 | SCAMP5 | secretory carrier membrane protein 5 |
| 299 | PDLIM5 | PDZ and LIM domain 5 | 328 | SERP1 | stress associated endoplasmic reticulum protein 1 |
| 300 | SRPK2 | SRSF protein kinase 2 | 329 | SNX16 | sorting nexin 16 |
| 301 | STAT3 | signal transducer and activator of transcription 3 | 330 | SOX4 | SRY-box 4 |
| 302 | WDFY2 | WD repeat and FYVE domain containing 2 | 331 | TMX3 | thioredoxin related transmembrane protein 3 |
| 303 | ZFPM2 | zinc finger protein, FOG family member 2 | 332 | TSG101 | tumor susceptibility 101 |
| 304 | ZNF236 | zinc finger protein 236 | 333 | WFS1 | wolframin ER transmembrane glycoprotein |
| 305 | ABI1 | abl interactor 1 | 334 | ZBTB21 | zinc finger and BTB domain containing 21 |
| 306 | AHNAK | AHNAK nucleoprotein | 335 | ZNF2 | zinc finger protein 2 |
| 307 | AKAP13 | A-kinase anchoring protein 13 | 336 | FAM13C | family with sequence similarity 13 member C |
| 308 | BAMBI | BMP and activin membrane bound inhibitor | 337 | IKZF4 | IKAROS family zinc finger 4 |
| 309 | BHLHE41 | basic helix-loop-helix family member e41 | 338 | MAPK4 | mitogen-activated protein kinase 4 |
| 310 | CDC37L1 | cell division cycle 37 like 1 | 339 | PLAG1 | PLAG1 zinc finger |
| 311 | CEP170 | centrosomal protein 170 | 340 | PPP3R1 | protein phosphatase 3 regulatory subunit B, alpha |
| 312 | CMPK1 | cytidine/uridine monophosphate kinase 1 | 341 | PTPDC1 | protein tyrosine phosphatase domain containing 1 |
| 313 | CNOT7 | CCR4-NOT transcription complex subunit 7 | 342 | RNF6 | ring finger protein 6 |
| 314 | CREB5 | cAMP responsive element binding protein 5 | 343 | SLC30A7 | solute carrier family 30 member 7 |
| 315 | HLF | HLF, PAR bZIP transcription factor | 344 | TSPAN9 | tetraspanin 9 |
| 316 | LMO3 | LIM domain only 3 | 345 | UXS1 | UDP-glucuronate decarboxylase 1 |
| 317 | LYPD6 | LY6/PLAUR domain containing 6 | 346 | ARHGEF3 | Rho guanine nucleotide exchange factor 3 |
| **No.** | **Gene Symbol** | **Gene Description** | **No.** | **Gene Symbol** | **Gene Description** |
| 347 | BICD2 | BICD cargo adaptor 2 | 376 | TRIM37 | tripartite motif containing 37 |
| 348 | C6orf120 | chromosome 6 open reading frame 120 | 377 | CDKN1A | cyclin dependent kinase inhibitor 1A |
| 349 | LCOR | ligand dependent nuclear receptor corepressor | 378 | CNOT6L | CCR4-NOT transcription complex subunit 6 like |
| 350 | MAP7 | microtubule associated protein 7 | 379 | EREG | epiregulin |
| 351 | MCF2L | MCF.2 cell line derived transforming sequence like | 380 | GABBR2 | gamma-aminobutyric acid type B receptor subunit 2 |
| 352 | NEUROG1 | neurogenin 1 | 381 | GNS | glucosamine (N-acetyl)-6-sulfatase |
| 353 | NHLRC3 | NHL repeat containing 3 | 382 | KCNJ10 | potassium voltage-gated channel subfamily J member 10 |
| 354 | PRR16 | proline rich 16 | 383 | KLF12 | Kruppel like factor 12 |
| 355 | PTPN21 | protein tyrosine phosphatase, non-receptor type 21 | 384 | NEUROG2 | neurogenin 2 |
| 356 | SH3BP5 | SH3 domain binding protein 5 | 385 | NFIB | nuclear factor I B |
| 357 | SLITRK2 | SLIT and NTRK like family member 2 | 386 | PAFAH1B1 | platelet activating factor acetylhydrolase 1b regulatory subunit 1 |
| 358 | SOCS6 | suppressor of cytokine signaling 6 | 387 | PLCB1 | phospholipase C beta 1 |
| 359 | ST6GALNAC3 | ST6 N-acetylgalactosaminide alpha-2,6-sialyltransferase 3 | 388 | PLS1 | plastin 1 |
| 360 | TBC1D8B | TBC1 domain family member 8B | 389 | SORL1 | sortilin related receptor 1 |
| 361 | TENM1 | teneurin transmembrane protein 1 | 390 | SPTY2D1 | SPT2 chromatin protein domain containing 1 |
| 362 | ZBTB8A | zinc finger and BTB domain containing 8A | 391 | ZDHHC9 | zinc finger DHHC-type containing 9 |
| 363 | ZNF25 | zinc finger protein 25 | 392 | ZNF280B | zinc finger protein 280B |
| 364 | ABHD2 | abhydrolase domain containing 2 | 393 | CPEB3 | cytoplasmic polyadenylation element binding protein 3 |
| 365 | BCL2L11 | BCL2 like 11 | 394 | KLF9 | Kruppel like factor 9 |
| 366 | C2orf69 | chromosome 2 open reading frame 69 | 395 | NDEL1 | nudE neurodevelopment protein 1 like 1 |
| 367 | DNAJB9 | DnaJ heat shock protein family (Hsp40) member B9 | 396 | NPAS3 | neuronal PAS domain protein 3 |
| 368 | MTF1 | metal regulatory transcription factor 1 | 397 | PHLPP2 | PH domain and leucine rich repeat protein phosphatase 2 |
| 369 | MYNN | myoneurin | 398 | RAB30 | RAB30, member RAS oncogene family |
| 370 | PARD6B | par-6 family cell polarity regulator beta | 399 | SCN2A | sodium voltage-gated channel alpha subunit 2 |
| 371 | PFKFB3 | 6-phosphofructo-2-kinase/fructose-2,6-biphosphatase 3 | 400 | SLC4A4 | solute carrier family 4 member 4 |
| 372 | POLR3G | RNA polymerase III subunit G | 401 | SOWAHC | sosondowah ankyrin repeat domain family member C |
| 373 | RBL1 | RB transcriptional corepressor like 1 | 402 | TAOK1 | TAO kinase 1 |
| 374 | SAMD8 | sterile alpha motif domain containing 8 | 403 | AKT3 | AKT serine/threonine kinase 3 |
| 375 | TMEM25 | transmembrane protein 25 | 404 | ATG14 | autophagy related 14 |
| **No.** | **Gene Symbol** | **Gene Description** | **No.** | **Gene Symbol** | **Gene Description** |
| 405 | ATP1A2 | ATPase Na+/K+ transporting subunit alpha 2 | 434 | M6PR | mannose-6-phosphate receptor, cation dependent |
| 406 | ATXN7L1 | ataxin 7 like 1 | 435 | PAFAH1B2 | platelet activating factor acetylhydrolase 1b catalytic subunit 2 |
| 407 | BTG2 | BTG anti-proliferation factor 2 | 436 | PLEKHO2 | pleckstrin homology domain containing O2 |
| 408 | FAM3C | family with sequence similarity 3 member C | 437 | PTGFRN | prostaglandin F2 receptor inhibitor |
| 409 | ITCH | itchy E3 ubiquitin protein ligase | 438 | SMIM14 | small integral membrane protein 14 |
| 410 | JAK1 | Janus kinase 1 | 439 | TBC1D15 | TBC1 domain family member 15 |
| 411 | KIAA1522 | KIAA1522 | 440 | TMBIM6 | transmembrane BAX inhibitor motif containing 6 |
| 412 | KPNA3 | karyopherin subunit alpha 3 | 441 | ACBD5 | acyl-CoA binding domain containing 5 |
| 413 | PPARA | peroxisome proliferator activated receptor alpha | 442 | CRIM1 | cysteine rich transmembrane BMP regulator 1 |
| 414 | SLC41A1 | solute carrier family 41 member 1 | 443 | MAP3K5 | mitogen-activated protein kinase kinase kinase 5 |
| 415 | SS18L1 | SS18L1, nBAF chromatin remodeling complex subunit | 444 | PHTF2 | putative homeodomain transcription factor 2 |
| 416 | STYX | serine/threonine/tyrosine interacting protein | 445 | UBE2J1 | ubiquitin conjugating enzyme E2 J1 |
| 417 | APBB2 | amyloid beta precursor protein binding family B member 2 | 446 | ARL4C | ADP ribosylation factor like GTPase 4C |
| 418 | ATXN1 | ataxin 1 | 447 | CCSER2 | coiled-coil serine rich protein 2 |
| 419 | COX7A2L | cytochrome c oxidase subunit 7A2 like | 448 | CREBRF | CREB3 regulatory factor |
| 420 | CXCL14 | C-X-C motif chemokine ligand 14 | 449 | CRIPT | CXXC repeat containing interactor of PDZ3 domain |
| 421 | EGLN1 | egl-9 family hypoxia inducible factor 1 | 450 | HMGA2 | high mobility group AT-hook 2 |
| 422 | ESR1 | estrogen receptor 1 | 451 | IPO9 | importin 9 |
| 423 | HOOK3 | hook microtubule tethering protein 3 | 452 | JAZF1 | JAZF zinc finger 1 |
| 424 | SGTB | small glutamine rich tetratricopeptide repeat containing beta | 453 | KLHL20 | kelch like family member 20 |
| 425 | ST8SIA2 | ST8 alpha-N-acetyl-neuraminide alpha-2,8-sialyltransferase 2 | 454 | PCMTD1 | protein-L-isoaspartate (D-aspartate) O-methyltransferase domain containing 1 |
| 426 | TWF1 | twinfilin actin binding protein 1 | 455 | PGP | phosphoglycolate phosphatase |
| 427 | UBE2B | ubiquitin conjugating enzyme E2 B | 456 | PURA | purine rich element binding protein A |
| 428 | ZNF362 | zinc finger protein 362 | 457 | RABEP1 | rabaptin, RAB GTPase binding effector protein 1 |
| 429 | CELSR2 | cadherin EGF LAG seven-pass G-type receptor 2 | 458 | STX6 | syntaxin 6 |
| 430 | CIT | citron rho-interacting serine/threonine kinase | 459 | TMEM50B | transmembrane protein 50B |
| 431 | EEA1 | early endosome antigen 1 | 460 | TTC39C | tetratricopeptide repeat domain 39C |
| 432 | ERI1 | exoribonuclease 1 | 461 | WEE1 | WEE1 G2 checkpoint kinase |
| 433 | KDM2A | lysine demethylase 2A | 462 | KLF10 | Kruppel like factor 10 |
| **No.** | **Gene Symbol** | **Gene Description** | **No.** | **Gene Symbol** | **Gene Description** |
| 463 | LRP1B | LDL receptor related protein 1B | 492 | ITFG1 | integrin alpha FG-GAP repeat containing 1 |
| 464 | MAPRE1 | microtubule associated protein RP/EB family member 1 | 493 | MAT2B | methionine adenosyltransferase 2B |
| 465 | NR4A2 | nuclear receptor subfamily 4 group A member 2 | 494 | PTPRJ | protein tyrosine phosphatase, receptor type J |
| 466 | RAPGEF4 | Rap guanine nucleotide exchange factor 4 | 495 | SHANK2 | SH3 and multiple ankyrin repeat domains 2 |
| 467 | RASGEF1A | RasGEF domain family member 1A | 496 | TSC22D2 | TSC22 domain family member 2 |
| 468 | RNF38 | ring finger protein 38 | 497 | CADM2 | cell adhesion molecule 2 |
| 469 | SHOC2 | SHOC2, leucine rich repeat scaffold protein | 498 | CSGALNACT1 | chondroitin sulfate N-acetylgalactosaminyltransferase 1 |
| 470 | TXLNA | taxilin alpha | 499 | FMNL3 | formin like 3 |
| 471 | BECN1 | beclin 1 | 500 | MPDZ | multiple PDZ domain crumbs cell polarity complex component |
| 472 | GPC6 | glypican 6 | 501 | PLSCR4 | phospholipid scramblase 4 |
| 473 | PKN2 | protein kinase N2 | 502 | SASH1 | SAM and SH3 domain containing 1 |
| 474 | PRRX1 | paired related homeobox 1 | 503 | TCF7L1 | transcription factor 7 like 1 |
| 475 | RNF2 | ring finger protein 2 | 504 | YPEL2 | yippee like 2 |
| 476 | E2F3 | E2F transcription factor 3 | 505 | CERS6 | ceramide synthase 6 |
| 477 | EIF4G2 | eukaryotic translation initiation factor 4 gamma 2 | 506 | CYB561D1 | cytochrome b561 family member D1 |
| 478 | GID4 | GID complex subunit 4 homolog | 507 | DCUN1D3 | defective in cullin neddylation 1 domain containing 3 |
| 479 | LHX8 | LIM homeobox 8 | 508 | SLC11A1 | solute carrier family 11 member 1 |
| 480 | NR4A3 | nuclear receptor subfamily 4 group A member 3 | 509 | TIMP2 | TIMP metallopeptidase inhibitor 2 |
| 481 | PFN2 | profilin 2 | 510 | TTPAL | alpha tocopherol transfer protein like |
| 482 | TP53INP1 | tumor protein p53 inducible nuclear protein 1 | 511 | ZNF217 | zinc finger protein 217 |
| 483 | TSHZ3 | teashirt zinc finger homeobox 3 | 512 | CHD9 | chromodomain helicase DNA binding protein 9 |
| 484 | ZBTB6 | zinc finger and BTB domain containing 6 | 513 | GMCL1 | germ cell-less, spermatogenesis associated 1 |
| 485 | ZC3H7B | zinc finger CCCH-type containing 7B | 514 | HDAC4 | histone deacetylase 4 |
| 486 | ARHGAP35 | Rho GTPase activating protein 35 | 515 | SLC25A36 | solute carrier family 25 member 36 |
| 487 | BMP2 | bone morphogenetic protein 2 | 516 | SMAD7 | SMAD family member 7 |
| 488 | CACUL1 | CDK2 associated cullin domain 1 | 517 | SNX21 | sorting nexin family member 21 |
| 489 | CEP128 | centrosomal protein 128 | 518 | SOBP | sine oculis binding protein homolog |
| 490 | HMGB3 | high mobility group box 3 | 519 | THBS2 | thrombospondin 2 |
| 491 | HP1BP3 | heterochromatin protein 1 binding protein 3 | 520 | CDCA7 | cell division cycle associated 7 |
| **No.** | **Gene Symbol** | **Gene Description** | **No.** | **Gene Symbol** | **Gene Description** |
| 521 | CYP26B1 | cytochrome P450 family 26 subfamily B member 1 | 550 | FBXO28 | F-box protein 28 |
| 522 | DGKH | diacylglycerol kinase eta | 551 | IL17RD | interleukin 17 receptor D |
| 523 | RSBN1 | round spermatid basic protein 1 | 552 | KMT2C | lysine methyltransferase 2C |
| 524 | SMAD1 | SMAD family member 1 | 553 | MECP2 | methyl-CpG binding protein 2 |
| 525 | SRGAP3 | SLIT-ROBO Rho GTPase activating protein 3 | 554 | MID1 | midline 1 |
| 526 | ADAMTS5 | ADAM metallopeptidase with thrombospondin type 1 motif 5 | 555 | PKIA | cAMP-dependent protein kinase inhibitor alpha |
| 527 | GIT2 | GIT ArfGAP 2 | 556 | CCND2 | cyclin D2 |
| 528 | IGF2BP1 | insulin like growth factor 2 mRNA binding protein 1 | 557 | FRMD4A | FERM domain containing 4A |
| 529 | MCC | MCC, WNT signaling pathway regulator | 558 | MED17 | mediator complex subunit 17 |
| 530 | MKLN1 | muskelin 1 | 559 | RND3 | Rho family GTPase 3 |
| 531 | PRDM10 | PR/SET domain 10 | 560 | CHIC1 | cysteine rich hydrophobic domain 1 |
| 532 | RAB11FIP4 | RAB11 family interacting protein 4 | 561 | DAZAP2 | DAZ associated protein 2 |
| 533 | ZRANB1 | zinc finger RANBP2-type containing 1 | 562 | EIF4H | eukaryotic translation initiation factor 4H |
| 534 | EPHB4 | EPH receptor B4 | 563 | FGF5 | fibroblast growth factor 5 |
| 535 | METAP1 | methionyl aminopeptidase 1 | 564 | FNBP4 | formin binding protein 4 |
| 536 | NETO2 | neuropilin and tolloid like 2 | 565 | HECA | hdc homolog, cell cycle regulator |
| 537 | PPP6R3 | protein phosphatase 6 regulatory subunit 3 | 566 | SDC2 | syndecan 2 |
| 538 | VEGFA | vascular endothelial growth factor A | 567 | TMEM123 | transmembrane protein 123 |
| 539 | YTHDF3 | YTH N6-methyladenosine RNA binding protein 3 | 568 | UBR5 | ubiquitin protein ligase E3 component n-recognin 5 |
| 540 | ARL1 | ADP ribosylation factor like GTPase 1 | 569 | YES1 | YES proto-oncogene 1, Src family tyrosine kinase |
| 541 | NOL4 | nucleolar protein 4 | 570 | FOXJ2 | forkhead box J2 |
| 542 | PKNOX1 | PBX/knotted 1 homeobox 1 | 571 | NUFIP2 | nuclear FMR1 interacting protein 2 |
| 543 | PPP2R2A | protein phosphatase 2 regulatory subunit Balpha | 572 | TBL1X | transducin beta like 1 X-linked |
| 544 | SLC25A27 | solute carrier family 25 member 27 | 573 | AJUBA | ajuba LIM protein |
| 545 | TNFSF11 | TNF superfamily member 11 | 574 | CDC40 | cell division cycle 40 |
| 546 | TRAPPC10 | trafficking protein particle complex 10 | 575 | CYP2U1 | cytochrome P450 family 2 subfamily U member 1 |
| 547 | WNK1 | WNK lysine deficient protein kinase 1 | 576 | FXR1 | FMR1 autosomal homolog 1 |
| 548 | ZNF532 | zinc finger protein 532 | 577 | RGS4 | regulator of G protein signaling 4 |
| 549 | CABLES1 | Cdk5 and Abl enzyme substrate 1 | 578 | SEMA5A | semaphorin 5A |
| **No.** | **Gene Symbol** | **Gene Description** | **No.** | **Gene Symbol** | **Gene Description** |
| 579 | DEDD | death effector domain containing | 605 | RCOR1 | REST corepressor 1 |
| 580 | DIP2A | disco interacting protein 2 homolog A | 606 | SMAD6 | SMAD family member 6 |
| 581 | FAF2 | Fas associated factor family member 2 | 607 | TCF4 | transcription factor 4 |
| 582 | FNDC3A | fibronectin type III domain containing 3A | 608 | TMCC1 | transmembrane and coiled-coil domain family 1 |
| 583 | FOXA1 | forkhead box A1 | 609 | ELAVL2 | ELAV like RNA binding protein 2 |
| 584 | MXI1 | MAX interactor 1, dimerization protein | 610 | FGF12 | fibroblast growth factor 12 |
| 585 | NAV2 | neuron navigator 2 | 611 | GJA1 | gap junction protein alpha 1 |
| 586 | PIP4K2A | phosphatidylinositol-5-phosphate 4-kinase type 2 alpha | 612 | UBE2W | ubiquitin conjugating enzyme E2 W |
| 587 | STC1 | stanniocalcin 1 | 613 | IPCEF1 | interaction protein for cytohesin exchange factors 1 |
| 588 | SULF1 | sulfatase 1 | 614 | MAP3K3 | mitogen-activated protein kinase kinase kinase 3 |
| 589 | AFF4 | AF4/FMR2 family member 4 | 615 | SNX9 | sorting nexin 9 |
| 590 | COL19A1 | collagen type XIX alpha 1 chain | 616 | TAOK2 | TAO kinase 2 |
| 591 | ERBB3 | erb-b2 receptor tyrosine kinase 3 | 617 | ZFYVE16 | zinc finger FYVE-type containing 16 |
| 592 | MYCN | MYCN proto-oncogene, bHLH transcription factor | 618 | CAMK2N1 | calcium/calmodulin dependent protein kinase II inhibitor 1 |
| 593 | NRSN1 | neurensin 1 | 619 | CTDSPL2 | CTD small phosphatase like 2 |
| 594 | SKI | SKI proto-oncogene | 620 | PHF6 | PHD finger protein 6 |
| 595 | TLE4 | TLE family member 4, transcriptional corepressor | 621 | USP53 | ubiquitin specific peptidase 53 |
| 596 | RARB | retinoic acid receptor beta | 622 | NBL1 | NBL1, DAN family BMP antagonist |
| 597 | SIRPA | signal regulatory protein alpha | 623 | NCEH1 | neutral cholesterol ester hydrolase 1 |
| 598 | TMEM132B | transmembrane protein 132B | 624 | PCDHA9 | protocadherin alpha 9 |
| 599 | TMUB2 | transmembrane and ubiquitin like domain containing 2 | 625 | RNF145 | ring finger protein 145 |
| 600 | UBASH3B | ubiquitin associated and SH3 domain containing B | 626 | TSKU | tsukushi, small leucine rich proteoglycan |
| 601 | VPS26A | VPS26, retromer complex component A | 627 | KLF3 | Kruppel like factor 3 |
| 602 | CNN1 | calponin 1 | 628 | NTN1 | netrin 1 |
| 603 | INTS6 | integrator complex subunit 6 | 629 | XIAP | X-linked inhibitor of apoptosis |
| 604 | PPM1A | protein phosphatase, Mg2+/Mn2+ dependent 1A |  |  |  |
